# Relationship between blood pressure and intraocular pressure in the JPHC-NEXT eye study

**DOI:** 10.1038/s41598-022-22301-1

**Published:** 2022-10-19

**Authors:** Tomoyo Yasukawa, Akiko Hanyuda, Kazumasa Yamagishi, Kenya Yuki, Miki Uchino, Yoko Ozawa, Mariko Sasaki, Kazuo Tsubota, Norie Sawada, Kazuno Negishi, Shoichiro Tsugane, Hiroyasu Iso

**Affiliations:** 1 Department of Ophthalmology, Keio University School of Medicine, 35 Shinanomachi, Shinjuku-ku, Tokyo, 160-8582 Japan; 2 Division of Epidemiology, National Cancer Center Institute for Cancer Control, 5-1-1 Tsukiji, Chuo-ku, Tokyo, 104-0045 Japan; 3 Department of Public Health Medicine, Faculty of Medicine, and Health Services Research and Development Centre, University of Tsukuba, 1-1-1 Tennodai, Tsukuba, Ibaraki 305-8575 Japan; 4 Ibaraki Western Medical Centre, 555 Otsuka, Chikusei, Ibaraki 308-0813 Japan; 5 Department of Ophthalmology, St. Luke’s International Hospital, 9-1 Akashi-cho, Chuo-ku, Tokyo, 104-8560 Japan; 6 Tachikawa Hospital, 4-2-22 Nishiki-cho, Tachikawa, Tokyo 190-8531 Japan; 7 National Hospital Organization Tokyo Medical Center, 2-5-1 Higashigaoka Meguro-ku, Tokyo, 152-8902 Japan; 8 Tsubota Laboratory, Inc., 34-304 Shinanomachi, Shinjuku-ku, Tokyo, 160-0016 Japan; 9 Division of Cohort Research, National Cancer Center Institute for Cancer Control, 5-1-1 Tsukiji, Chuo-ku, Tokyo, 104-0045 Japan; 10 National Institute of Health and Nutrition, National Institutes of Biomedical Innovation, Health and Nutrition, 1-23-1 Toyama, Shinjuku-ku, Tokyo, 162-8636 Japan; 11 Department of Social and Environmental Medicine, Osaka University Graduate School of Medicine, 2-2 Yamadaoka, Suita, Osaka 565-0871 Japan

**Keywords:** Ocular hypertension, Optic nerve diseases

## Abstract

Although a positive link between hypertension and intraocular pressure (IOP) has been suggested, the individual effects of systolic and diastolic blood pressure (SBP and DBP, respectively) on IOP remain unclear, particularly among Japanese populations. Here, we conducted a large-scale, cross-sectional study to determine individual and combined effects of SBP/DBP and hypertension on IOP. In total, 6783 Japanese people aged over 40 years underwent systemic and ophthalmological examinations, including measurements of blood pressure and IOP, conducted using non-contact tonometers. After adjusting for a priori known confounding factors, SBP and DBP levels were found to be positively correlated with IOP levels. The multivariable-adjusted odds ratio when comparing the hypertensive and normotensive groups for the prevalence of ocular hypertension was 1.88 (95% confidence interval, 1.14–3.08). When analysing the combined effects of SBP and DBP on ocular hypertension, SBP elevation had a greater effect on ocular hypertension than DBP increase. In conclusion, SBP and DBP levels and the prevalence of systemic hypertension were found to be positively associated with IOP levels and the prevalence of ocular hypertension in an ophthalmologically healthy Japanese population. Our findings suggest that systemic blood pressure control may be key for controlling IOP.

## Introduction

Intraocular pressure (IOP) is determined by the balance between aqueous humour production and outflow, and IOP homeostasis is primarily maintained by changes in aqueous humour outflow resistance^1^. Epidemiological studies have suggested that IOP is affected by several factors, including non-modifiable risk factors, such as age, race, refraction, and central corneal thickness (CCT), and modifiable risk factors, such as blood pressure (BP), physical activity, and obesity^2–5^. Elevated IOP may cause glaucomatous optic nerve damage and subsequent visual field deficits^6^, leading to substantial limitations in daily functioning and loss of autonomy^7^. Therefore, exploring modifiable lifestyle factors related to IOP is of significant importance.

Several epidemiological studies have suggested a positive correlation between systemic BP and IOP^8^; however, the differential effect of systolic vs. diastolic BP (SBP vs. DBP) on IOP remains unclear. Although the population mean systemic BP in Japan has decreased substantially over the past decades^9^, hypertension remains the highest preventable cause of cardiovascular diseases^10^. Furthermore, the independent burdens of systolic and diastolic hypertension on cardiovascular events are increasingly being recognised^11^. Therefore, it is important to examine the differential effects of SBP and DBP on IOP from the public health perspective as well.

According to a recent meta-analysis, 13 cross-sectional and four longitudinal studies reported that a 10-mmHg increase in SBP was associated with an average increase of 0.26 mmHg (95% confidence interval [CI] 0.23–0.28) in IOP, and 10 cross-sectional and four longitudinal studies reported that a 5-mmHg increase in DBP was associated with an average increase of 0.17 mmHg (95% CI 0.11–0.23) in IOP^8^. Many previous studies have also reported a positive association between BP levels and IOP but have not accounted for several confounding factors^12,13^. For instance, individuals with hypertension often have systemic lifestyle-related diseases, such as diabetes, dyslipidaemia, and obesity, which are known to be related to IOP^14–16^. Moreover, an association between the use of antihypertensive medication and IOP has also been reported^17^. The few population-based studies in Japan examining the association between BP levels and IOP are similar to those mentioned above and were conducted using data obtained prior to 2010^18–20^.

Therefore, the purpose of our study was to examine the individual as well as combined effects of SBP and DBP on IOP after comprehensively adjusting for potential confounding factors and using the largest dataset of relatively healthy Japanese population till date.

## Results

### Baseline characteristics of participants according to hypertensive status

**Figure 1:**
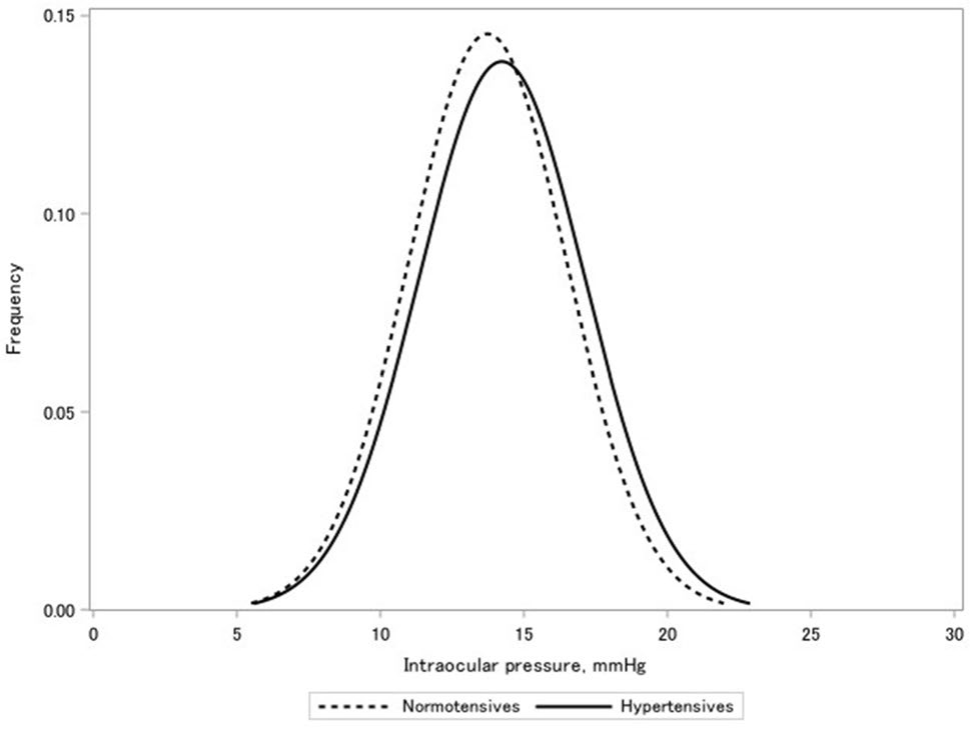
Distribution of intraocular pressure according to hypertensive status.

Of the 6783 participants included in the study, 3152 (46.5%; 1540 men and 1612 women) had hypertension. The baseline characteristics of participants according to hypertensive status are presented in Table 1. Compared to participants without hypertension, those with hypertension were older and more likely to have increased CCT; lower waist circumference and high-density lipoprotein cholesterol (HDL-C) and low-density lipoprotein cholesterol (LDL-C) levels; and higher body mass index (BMI), alcohol intake, and triglyceride and glycated haemoglobin (HbA1c) levels. Furthermore, the mean IOP ± standard deviation (SD) was higher in participants with hypertension (14.3 ± 2.8 mmHg) than in those without hypertension (13.8 ± 2.6 mmHg). The distribution of IOP according to hypertensive status is shown in Fig. 1. Similarly, the mean SBP ± SD and DBP ± SD were higher in participants with hypertension (138.9 ± 16.6 mmHg and 80.6 ± 11.4 mmHg, respectively) than in those without hypertension (118.3 ± 12.2 mmHg and 70.8 ± 9.1 mmHg, respectively).

**Table 1:** Baseline demographic and clinical characteristics of included patients according to hypertensive status.

Characteristics	Non-hypertension (n = 3631)	Hypertension^b^ (n = 3152)
Age in years (SD)	61.0 (10.3)	66.8 (8.3)
Men, n (%)	66.1	51.1
Body mass index, kg/m^2^ (SD)	22.4 (3.1)	24.1 (3.3)
HDL cholesterol, mg/dL (SD)	65.2 (16.0)	60.8 (15.3)
Triglyceride, mg/dL (SD)	105.8 (64.8)	123.7 (74.2)
HbA1c, % (SD)	5.7 (0.6)	5.9 (0.7)
LDL cholesterol, mg/dL (SD)	127.9 (32.5)	122.3 (29.6)
Waist circumference, cm (SD)	75.7 (21.1)	73.4 (31.0)
**Fasting status**		
Non-fasting, %	32.4	37.0
Fasting, %	67.6	63.0
Diabetes, n (%)^a^	7.0	15.3
Systolic blood pressure, mmHg (SD)	118.3 (12.2)	138.9 (16.6)
Diastolic blood pressure, mmHg (SD)	70.8 (9.1)	80.6 (11.4)
**Alcohol intake, n (%)**		
Never, %	60.8	54.9
Sometimes, %	17.7	14.4
Current, %	21.5	30.6
**Smoking status, n (%)**		
Never, %	65.4	57.0
Former, %	20.9	30.8
Current, %	13.6	12.2
Average intraocular pressure in both eyes, mmHg (SD)	13.8 (2.6)	14.3 (2.8)
Average central corneal thickness in both eyes, µm (SD)	549.7 (58.9)	551.3 (57.7)

Values are presented as the means (SDs) for continuous variables and percentages for categorical variables.

HbA1c, haemoglobin A1c; HDL, high-density lipoprotein; LDL, low-density lipoprotein; SD, standard deviation.

^a^Diabetes was defined as self-reported use of antidiabetic medication, self-reported history of diabetes, or HbA1c ≥ 6.5%

^b^Hypertension was defined as use of any antihypertensive medication, systolic blood pressure ≥ 140 mmHg, and/or diastolic blood pressure ≥ 90 mmHg.

### Associations of hypertension, SBP, and DBP with IOP

After adjusting for age and sex, the prevalence of hypertension was found to be positively associated with IOP levels. The mean IOP ± standard error (SE) was 14.5 ± 0.1 mmHg for participants with hypertension and 13.7 ± 0.1 mmHg for those without hypertension (P < 0.001) (Table 2). When SBP and DBP were classified into five categories, there was a statistically significant trend toward increased IOP in participants with higher categories of SBP and DBP (P for the trend < 0.001 for both).

**Table 2:** Adjusted mean intraocular pressure according to systolic and diastolic blood pressures and hypertensive status.

Characteristics	No. of cases	Mean intraocular pressure, mmHg (SE)			
		Age- and sex-adjusted	P_trend_	Multivariable adjusted^a^	P_trend_
**Systolic blood pressure, mmHg**					
< 120	2263	13.3 (0.1)	< 0.001	13.4 (0.1)	< 0.001
120–129	1506	14.0 (0.1)		14.0 (0.1)	
130–139	1377	14.4 (0.1)		14.3 (0.1)	
140–159	1355	14.7 (0.1)		14.5 (0.1)	
≥ 160	282	15.7 (0.2)		15.4 (0.2)	
Per 10-mmHg increase	6783	0.38 (0.0)	< 0.001	0.32 (0.0)	< 0.001
**Diastolic blood pressure, mmHg**					
< 80	4469	13.8 (0.1)	< 0.001	13.7 (0.1)	< 0.001
80–84	952	14.5 (0.1)		14.4 (0.1)	
85–89	670	14.6 (0.1)		14.4 (0.1)	
90–99	559	14.6 (0.1)		14.5 (0.1)	
≥ 100	133	15.4 (0.2)		15.1 (0.2)	
Per 10-mmHg increase	6783	0.46 (0.0)	< 0.001	0.41 (0.0)	< 0.001
**Hypertension**^**b**^					
No	3631	13.7 (0.1)	< 0.001	13.7 (0.1)	< 0.001
Yes	3152	14.5 (0.1)		14.4 (0.1)	

LDL, low-density lipoprotein; SE, standard error.

^a^Adjusted for age, sex, smoking status, alcohol intake, diabetes, body mass index, LDL-C level, and central corneal thickness. For systolic blood pressure and diastolic blood pressure, we have additionally adjusted for use of any antihypertensive medication.

^b^Hypertension was defined as use of any antihypertensive medication, systolic blood pressure ≥ 140 mmHg, and/or diastolic blood pressure ≥ 90 mmHg.

After further adjusting for smoking status, alcohol intake, diabetes, BMI, LDL-C level, and CCT, the prevalence of hypertension remained positively associated with IOP levels. The mean IOP ± SE was 14.4 ± 0.1 mmHg for participants with hypertension and 13.7 ± 0.1 mmHg for those without hypertension (P < 0.001). We observed a similar significant trend toward increased IOP in participants with higher categories of SBP and DBP in the multivariable-adjusted models (P for the trend < 0.001 for both). With every 10-mmHg increase in SBP/DBP, the IOP increased by 0.32/0.41 mmHg. When separately examined by sex, the positive correlations of hypertension, SBP, and DBP with IOP were generally consistent in both women and men (Supplementary Table S1a,b). In exploratory analyses, we further conducted stratified analyses by antihypertensive medication use (Supplementary Table S2); the positive association between BP levels and IOP was generally consistent, irrespective of medication status.

### Associations of hypertension, SBP, and DBP with ocular hypertension

After adjusting for age and sex, participants with hypertension had a higher prevalence of ocular hypertension than those without hypertension (odds ratio [OR], 2.02; 95% CI 1.36–3.02) (Table 3). When SBP and DBP were classified into five categories, participants with the highest categories of SBP and DBP had a higher prevalence of ocular hypertension than those with the lowest categories of SBP and DBP (OR, 5.71; 95% CI 2.79*–*11.7 and OR, 4.00; 95% CI 1.86–8.59, respectively). There was a statistically significant trend toward increasing OR for the prevalence of ocular hypertension in participants with higher categories of SBP and DBP (P for the trend < 0.001 for both).

**Table 3:** Odds ratios of ocular hypertension according to systolic and diastolic blood pressures and hypertensive status.

Characteristics	No. of cases	Odds ratio (95% confidence interval)			
		Age- and sex-adjusted	P_trend_	Multivariable adjusted^a^	P_trend_
**Systolic blood pressure, mmHg**					
< 120	19	1 (reference)	< 0.001	1 (reference)	< 0.001
120–129	23	1.83 (0.99–3.38)		1.99 (0.96–4.09)	
130–139	26	2.27 (1.25–4.12)		2.09 (0.99–4.39)	
140–159	34	3.04 (1.73–5.35)		3.48 (1.72–7.04)	
≥ 160	13	5.71 (2.79–11.7)		5.19 (2.05–13.1)	
**Diastolic blood pressure, mmHg**					
< 80	67	1 (reference)	< 0.001	1 (reference)	< 0.001
80–84	16	1.15 (0.66–1.99)		1.18 (0.62–2.24)	
85–89	8	0.82 (0.39–1.71)		0.83 (0.35–1.99)	
90–99	16	1.93 (1.11–3.37)		1.95 (0.98–3.90)	
≥ 100	8	4.00 (1.86–8.59)		4.28 (1.80–10.2)	
**Hypertension**^**b**^					
No	47	1 (reference)	< 0.001	1 (reference)	0.01
Yes	68	2.02 (1.36–3.02)		1.88 (1.14–3.08)	

LDL, low-density lipoprotein.

^a^Adjusted for age, sex, smoking status, alcohol intake, diabetes, body mass index, LDL-C level, and central corneal thickness. For systolic blood pressure and diastolic blood pressure, we have additionally adjusted for use of any antihypertensive medication.

^b^Hypertension was defined as use of any antihypertensive medication, systolic blood pressure ≥ 140 mmHg, and/or diastolic blood pressure ≥ 90 mmHg.

After further adjusting for smoking status, alcohol intake, diabetes, BMI, LDL-C level, and CCT, the prevalence of hypertension was found to be positively associated with the prevalence of ocular hypertension (multivariable-adjusted OR, 1.88; 95% CI 1.14–3.08). Similarly, we observed a positive association between higher SBP and DBP categories and a higher prevalence of ocular hypertension (multivariable-adjusted OR, 5.19; 95% CI 2.05–13.1 for SBP ≥ 160 mmHg vs. < 120 mmHg and multivariable-adjusted OR, 4.28; 95% CI 1.80–10.2 for DBP ≥ 100 mmHg vs. < 80 mmHg). There was a similar statistically significant trend toward increasing OR for the prevalence of ocular hypertension in participants with higher categories of SBP and DBP in the multivariable-adjusted models (P for the trend < 0.001 and < 0.001, respectively). The positive trend for BP levels and the prevalence of ocular hypertension was consistent, regardless of sex (Supplementary Table S3a,b) and antihypertensive medication use (Supplementary Table S4). In the exploratory analysis, we further examined the combined effects of systolic and diastolic hypertension on the prevalence of ocular hypertension. Compared with individuals who had no hypertension (SBP < 120 mmHg and DBP < 80 mmHg), those with systolic hypertension had over 17 times higher odds of ocular hypertension (multivariable-adjusted OR, 17.4; 95% CI 3.51–86.2 for SBP ≥ 160 mmHg and DBP < 80 mmHg vs. SBP < 120 mmHg and DBP < 80 mmHg) (Supplementary Table  S5). Although the association was attenuated, there was a non-significant positive association between diastolic hypertension and ocular hypertension.

## Discussion

In this cross-sectional study, we examined the association between BP levels and IOP in an ophthalmologically normal Japanese population. After adjusting for several confounding factors, we observed that SBP and DBP levels and the prevalence of hypertension were positively associated with IOP levels and the prevalence of ocular hypertension. Even after adjusting for antihypertensive medication use and CCT, there was no appreciable change in these associations.

Several cross-sectional^12,21,22^ and longitudinal studies^23,24^ have reported a positive association between SBP and IOP. Zhao et al. performed a meta-analysis of 60 studies, in which 13 cross-sectional studies and four longitudinal studies, including 12 community-based studies, were incorporated to evaluate the association between SBP and IOP^8^. All studies reported a positive association between SBP and IOP, and a 10-mmHg increase in SBP was found to be associated with a 0.26-mmHg (95% CI 0.23–0.28) increase in IOP, which was generally comparable to that in our current study (IOP [mean ± SE] increase of 0.32 ± 0.02 mmHg per 10-mmHg increase in SBP). McLeod et al.^25^ reported that SBP was 6.20 mmHg higher in individuals with ocular hypertension than in those without (P < 0.01). This also corresponded with our findings, in that participants with SBP ≥ 160 mmHg had a > 5-time higher prevalence of ocular hypertension than those with SBP < 120 mmHg. While only a few studies have assessed the association between BP levels and the prevalence of ocular hypertension, most have suggested a positive trend^26–28^, similar to our study. On the other hand, a clinic-based case–control study (91 cases and 91 controls) from Canada reported that individuals with ocular hypertension had an 80% lower prevalence of systemic hypertension^29^; these differences could be attributable to the smaller sample size, younger age (mean age, 40 years), or the comparison of treatment for systemic hypertension but not BP levels.

In this study, DBP was found to be positively associated with IOP and the prevalence of ocular hypertension—a 10-mmHg increase in DBP was related to a 0.41 ± 0.03-mmHg increase in IOP, and the prevalence of ocular hypertension was almost three times higher in those with DBP ≥ 100 mmHg than in those with DBP < 80 mmHg. To date, only a few studies have examined the association between DBP and IOP. Consistent with our study, two community-based studies showed a positive association between DBP and IOP^12,24^; no association was reported in some hospital-based studies^25,30^, although this could be due to the smaller sample sizes (n =  ~ 1000) or differences in study settings.

The pathogenesis underlying the relationship between BP and IOP remains unclear; however, several hypotheses have been proposed. Increased BP may lead to increased ultrafiltration of aqueous humour due to elevated ciliary artery pressure in the ciliary body^31^, as well as elevated episcleral venous pressure, which affects aqueous humour outflow^21^, resulting in increased IOP. Physiologic factors such as increased sympathetic tone or serum corticosteroid level in individuals with hypertension can also affect increased IOP; apart from BP, these factors may influence the association between DBP and IOP as well^32^. In the current study, SBP elevation had a greater effect on ocular hypertension than DBP. Shiose^33^ reported that high SBP may increase aqueous humour formation by ultrafiltration. Together with our current data, these lines of evidence suggest that the SBP peak wave reaching the eye may be a more essential determinant of IOP than the perfusion pressure expressed by DBP^33^.

Individuals with hypertension often have lifestyle diseases such as diabetes, dyslipidaemia, and obesity^14–16^, and the same trend was observed for the participants in our study (67% of participants with hypertension were taking antihypertensive medications). Moreover, some studies have reported that the use of β-blockers (a type of antihypertensive medication) is related to decreased IOP^17^. Therefore, we additionally examined whether the association between BP levels and IOP differed by antihypertensive medication use and confirmed that the observed positive associations were generally consistent irrespective of medication use status. Collectively, our current results provided robust evidence regarding the positive link between systemic BP levels and IOP.

The strengths of this study include its large sample size, the use of validated questionnaires to gather information regarding lifestyle and medical history, and adjustments for major confounders for the examined associations. Moreover, experienced ophthalmologists evaluated the ocular parameters based on the ocular examinations.

However, our study has several limitations as well. First, we cannot infer causality due to the cross-sectional study design. Nonetheless, there is limited population-based evidence with regard to the association between systemic BP levels and IOP. Therefore, we believe that this large-scale study with substantial lifestyle-related data is still important. Second, IOP was measured using the non-contact tonometer instead of the Goldmann applanation tonometer, which is considered the most precise and reliable tool for measuring IOP^34^. However, in this large population-based setting, IOP measurement using a non-contact tonometer was less invasive and reduced the inter-investigator heterogeneity, leading to more precise estimates and higher participation rate. In addition, the mean IOP ± SD of 14.1 ± 2.9 mmHg in this study was close to that of 14.5 mmHg obtained using the Goldmann applanation tonometer for 3021 non-glaucomatous Japanese individuals aged over 40 years in the Tajimi Study^19^. Third, because IOP was not measured under the same conditions in all participants, it may be influenced by diurnal variation, seasonal variation, and consumption of food and beverages^35–37^. Fourth, we did not conduct slit-lamp microscopy or gonioscopy examinations. Considering that anterior chamber depth and angle widths are important parameters for assessing IOP^38^, further studies are warranted.

In conclusion, we showed that SBP and DBP levels and the prevalence of hypertension were positively associated with IOP levels and the prevalence of ocular hypertension in an ophthalmologically healthy Japanese population. These findings suggest that higher systemic BP might influence IOP elevation.

## Methods

### Study population

The Japan Public Health Center-based Prospective Study for the Next Generation (JPHC-NEXT) Eye Study is an ancillary study of the JPHC-NEXT^39^. In total, 9940 individuals participated in the JPHC-NEXT between 2013 and 2017. Among those individuals, we excluded those who did not undergo ocular examinations (n = 1,030); those who had a diagnosis of glaucoma or a history of any IOP-lowering treatment (n = 490); those who had a history of ocular surgery, including refractive or cataract surgeries, or ocular laser treatment for corneal oedema or dystrophy (n = 1051); and those who had missing or outlying IOP (the upper and lower 1% tiles of the total population) measurements (n = 586). The remaining 6783 participants (2771 men and 4012 women) were included in our analysis. Residents of Chikusei City, Ibaraki Prefecture, aged over 40 years, participated in the study. Included participants were asked about medical history of diabetes or hypertension, histories of ocular disease and related surgery, smoking and alcohol intake, and medication use through trained technician interviews or a self-administered questionnaire. They also underwent systemic and ophthalmological examinations.

Written informed consent was obtained from all individuals who participated in this study. The study was conducted in accordance with the Ethical Guidelines for Medical and Health Research Involving Human Subjects, Japan. It was approved by the Medical Ethics Committees of Keio University (Tokyo), University of Tsukuba (Ibaraki), Osaka University (Osaka), and the National Cancer Center (Tokyo).

### Systemic examination and BP measurement

BP was measured twice on the right upper arm while the participant was seated, and the mean value of the two measurements was used for this study. Height was measured with socks on, and weight was measured with light clothing on. BMI was calculated as weight (kg) divided by height (m) squared. Sera were collected to measure HbA1c (%), HDL-C (mg/dl), LDL-C (mg/dl), and triglyceride (mg/dl) levels. The participants were asked to come for the examination in a fasting state, although this was not mandatory. Approximately 34.5% of the participants had serum drawn in the non-fasting status (< 8 h from the last meal). Hypertension was defined as use of any antihypertensive medication, SBP ≥ 140 mmHg, and/or DBP ≥ 90 mmHg^40^. Diabetes was defined as self-reported use of antidiabetic medication, self-reported history of diabetes, or HbA1c ≥ 6.5%^41^.

### Ocular examination and IOP measurement

Ocular examinations, including IOP and CCT measurement, were conducted. IOP of both eyes was measured using a non-contact tonometer (Tonoref™ II; Nidek Co., Ltd., Aichi, Japan), and the mean value of three measurements of both eyes was used for this analysis. CCT of both eyes was measured using a specular-type pachymeter (Specular Microscope XIII; Konan, Nishinomiya, Japan). Ocular hypertension, which is known to be a pre-stage of glaucoma^42^, was defined as an average IOP > 21 mmHg in both eyes, excluding cases of optic disc abnormalities, those with a history of self-reported physician-diagnosed glaucoma, or use of any antiglaucoma therapy^43^. Such individuals were identified by trained technicians based on interviews or self-administered questionnaires.

### Statistical analysis

BP values were classified into five categories according to the 2018 European Society of Cardiology/European Society of Hypertension guidelines for the management of arterial hypertension—SBP (< 120, 120–129, 130–139, 140–159, and ≥ 160 mmHg) and DBP (< 80, 80–84, 85–89, 90–99, and ≥ 100 mmHg)^44^.

The associations of IOP with hypertension, SBP, and DBP were assessed using two analysis of covariance models. The first model was adjusted for age (continuous) and sex (men vs. women). The second model was further adjusted for smoking status (never, former, or current smokers), alcohol intake (never, sometimes, or current drinkers), diabetes (yes vs. no), BMI (< 25 vs. ≥ 25 kg/m^2^), LDL-C level (continuous), and CCT (continuous). Use of antihypertensive medication was additionally adjusted in the second model to determine the associations of SBP and DBP with IOP. The trend tests across the SBP/DBP categories were assigned to the median value for each category as a continuous variable and evaluated using the Wald test. In alternate analyses, the relation with a 10-mmHg increment of SBP/DBP was also evaluated. To consider the sex-disparity of ocular biometric features^45^, we additionally conducted sex-stratified analyses.

The associations of hypertension, SBP, and DBP with the prevalence of ocular hypertension were assessed using two multivariable logistic regression models. These were adjusted for the same confounding factors as the models to assess the associations of hypertension, SBP, and DBP with IOP. The results are expressed as ORs with 95% CIs. We assigned the median value for each SBP/DBP category and provided a P-value for the trend test. The lowest categories of SBP and DBP and participants without hypertension were used as the reference groups. The combined association of SBP and DBP with ocular hypertension was also evaluated. In a secondary analysis, we further stratified the analysis jointly by antihypertensive medication use to minimise the potential confounding effect of these variables.

All P values were two-sided, and a P-value < 0.05 was considered statistically significant. All analyses were performed using SAS software (Version 9.4, SAS Institute, Cary, NC, USA).

## Supplementary Information

Supplementary Tables.

## Data Availability

Information on how to access JPHC-NEXT data and/or biospecimens is available at https://epi.ncc.go.jp/jphcnext/en/access/index.html.
