# Supplementary material for: Relationship between blood pressure and intraocular pressure in the JPHC-NEXT eye study
Source: Sci Rep. 2022 Oct 19;12:17493. doi: 10.1038/s41598-022-22301-1 (PMC9582013; doi:10.1038/s41598-022-22301-1)
Supplement: Supplementary file 1 — Supplementary Tables. [file 41598_2022_22301_MOESM1_ESM.docx]

**Supplementary Information**

**Relationship Between Blood Pressure and Intraocular Pressure in the JPHC-NEXT Eye Study**

Tomoyo Yasukawa^1^, Akiko Hanyuda^1,2^*, Kazumasa Yamagishi^3,4^*, Kenya Yuki^1^, Miki Uchino^1^, Yoko Ozawa^1,5^, Mariko Sasaki^1,6,7^, Kazuo Tsubota^8^, Norie Sawada^9^, Kazuno Negishi^1^, Shoichiro Tsugane^9,10^, Hiroyasu Iso^11^

Supplementary Table S1a. Adjusted mean intraocular pressure (IOP) according to systolic and diastolic blood pressures and hypertensive status in men

| Characteristics | No. of cases | Mean IOP, mmHg (SE) | | | |
| --- | --- | --- | --- | --- | --- |
|  |  | Age-adjusted | P_trend_ | Multivariable-adjusted^†^ | P_trend_ |
| Systolic blood pressure, mmHg |  |  |  |  |  |
| <120 | 694 | 13.0 (0.1) | <0.001 | 13.1 (0.1) | <0.001 |
| 120–129 | 624 | 13.8 (0.1) |  | 13.8 (0.1) |  |
| 130–139 | 646 | 14.0 (0.1) |  | 14.0 (0.1) |  |
| 140–159 | 675 | 14.3 (0.1) |  | 14.1 (0.1) |  |
| ≥160 | 132 | 15.2 (0.2) |  | 15.0 (0.2) |  |
| Per 10-mmHg increase | 2771 | 0.34 (0.0) | <0.001 | 0.30 (0.0) | <0.001 |
| Diastolic blood pressure, mmHg |  |  |  |  |  |
| <80 | 1594 | 13.5 (0.1) | <0.001 | 13.5 (0.1) | <0.001 |
| 80–84 | 456 | 14.2 (0.1) |  | 14.2 (0.1) |  |
| 85–89 | 355 | 14.5 (0.1) |  | 14.4 (0.1) |  |
| 90–99 | 285 | 14.2 (0.1) |  | 14.1 (0.1) |  |
| ≥100 | 81 | 14.9 (0.2) |  | 14.6 (0.2) |  |
| Per 10-mmHg increase | 2771 | 0.45 (0.0) | <0.001 | 0.38 (0.0) | <0.001 |
| Hypertension^¶^ |  |  |  |  |  |
| No | 1231 | 13.5 (0.1) | <0.001 | 13.6 (0.1) | <0.001 |
| Yes | 1540 | 14.1 (0.1) |  | 14.0 (0.1) |  |

^†^Adjusted for age, smoking status, alcohol intake, diabetes, body mass index, LDL-C level, and central corneal thickness. For systolic blood pressure and diastolic blood pressure, we have additionally adjusted for use of any antihypertensive medication.

^¶^Hypertension was defined as use of any antihypertensive medication, systolic blood pressure ≥140 mmHg, and/or diastolic blood pressure ≥90 mmHg.

LDL, low-density lipoprotein; SE, standard error

Supplementary Table S1b. Adjusted mean intraocular pressure (IOP) according to systolic and diastolic blood pressures and hypertensive status in women

| Characteristics | No. of cases | Mean IOP, mmHg (SE) | | | |
| --- | --- | --- | --- | --- | --- |
|  |  | Age-adjusted | P_trend_ | Multivariable-adjusted^†^ | P_trend_ |
| Systolic blood pressure, mmHg |  |  |  |  |  |
| <120 | 1569 | 13.5 (0.1) | <0.001 | 13.5 (0.1) | <0.001 |
| 120–129 | 882 | 14.1 (0.1) |  | 14.1 (0.1) |  |
| 130–139 | 731 | 14.7 (0.1) |  | 14.5 (0.1) |  |
| 140–159 | 680 | 14.9 (0.1) |  | 14.7 (0.1) |  |
| ≥160 | 150 | 16.1 (0.2) |  | 15.8 (0.2) |  |
| Per 10-mmHg increase | 4012 | 0.41 (0.0) | <0.001 | 0.36 (0.0) | <0.001 |
| Diastolic blood pressure, mmHg |  |  |  |  |  |
| <80 | 2875 | 13.9 (0.1) | <0.001 | 13.8 (0.1) | <0.001 |
| 80–84 | 496 | 14.6 (0.1) |  | 14.4 (0.1) |  |
| 85–89 | 315 | 14.5 (0.1) |  | 14.3 (0.1) |  |
| 90–99 | 274 | 14.9 (0.1) |  | 14.8 (0.1) |  |
| ≥100 | 52 | 15.7 (0.2) |  | 15.6 (0.2) |  |
| Per 10-mmHg increase | 4012 | 0.47 (0.0) | <0.001 | 0.42 (0.0) | <0.001 |
| Hypertension^¶^ |  |  |  |  |  |
| No | 2400 | 13.7 (0.1) | <0.001 | 13.7 (0.1) | <0.001 |
| Yes | 1612 | 14.7 (0.1) |  | 14.5 (0.1) |  |

^†^Adjusted for age, smoking status, alcohol intake, diabetes, body mass index, LDL-C level, and central corneal thickness. For systolic blood pressure and diastolic blood pressure, we have additionally adjusted for use of any antihypertensive medication.

^¶^Hypertension was defined as use of any antihypertensive medication, systolic blood pressure ≥140 mmHg, and/or diastolic blood pressure ≥90 mmHg.

LDL, low-density lipoprotein; SE, standard error

Supplementary Table S2. Adjusted mean intraocular pressure (IOP) according to systolic and diastolic blood pressures, stratified by antihypertensive medication use

| Characteristics | No. of cases | Mean IOP, mmHg (SE) | | | |
| --- | --- | --- | --- | --- | --- |
|  |  | Age- and sex-adjusted | P_trend_ | Multivariable-adjusted^†^ | P_trend_ |
| **Participants not using antihypertensive medication (n = 4673)** | | | | | |
| Systolic blood pressure, mmHg |  |  |  |  |  |
| <120 | 1855 | 13.4 (0.1) | <0.001 | 13.4 (0.1) | <0.001 |
| 120–129 | 1038 | 14.0 (0.1) |  | 14.0 (0.1) |  |
| 130–139 | 828 | 14.4 (0.1) |  | 14.3 (0.1) |  |
| 140–159 | 785 | 14.7 (0.1) |  | 14.6 (0.1) |  |
| ≥160 | 167 | 15.9 (0.2) |  | 15.8 (0.2) |  |
| Diastolic blood pressure, mmHg |  |  |  |  |  |
| <80 | 3216 | 13.7 (0.05) | <0.001 | 13.7 (0.05) | <0.001 |
| 80–84 | 604 | 14.7 (0.1) |  | 14.4 (0.1) |  |
| 85–89 | 399 | 14.5 (0.1) |  | 14.4 (0.1) |  |
| 90–99 | 358 | 14.7 (0.1) |  | 14.5 (0.1) |  |
| ≥100 | 96 | 15.5 (0.3) |  | 15.3 (0.3) |  |
| **Participants using antihypertensive medication (n = 2110)** | | | | | |
| Systolic blood pressure, mmHg |  |  |  |  |  |
| <120 | 408 | 13.3 (0.1) | <0.001 | 13.5 (0.1) | <0.001 |
| 120–129 | 468 | 13.9 (0.1) |  | 13.9 (0.1) |  |
| 130–139 | 549 | 14.3 (0.1) |  | 14.2 (0.1) |  |
| 140–159 | 570 | 14.5 (0.1) |  | 14.3 (0.1) |  |
| ≥160 | 115 | 15.3 (0.3) |  | 14.9 (0.3) |  |
| Diastolic blood pressure, mmHg |  |  |  |  |  |
| <80 | 1253 | 13.8 (0.1) | <0.001 | 13.7 (0.1) | <0.001 |
| 80–84 | 348 | 14.5 (0.1) |  | 14.4 (0.1) |  |
| 85–89 | 271 | 14.7 (0.2) |  | 14.6 (0.2) |  |
| 90–99 | 201 | 14.5 (0.2) |  | 14.5 (0.2) |  |
| ≥100 | 37 | 14.7 (0.5) |  | 14.5 (0.5) |  |

^†^Adjusted for age, sex, smoking status, alcohol intake, diabetes, body mass index, LDL-cholesterol level, and central corneal thickness.

LDL, low-density lipoprotein; SE, standard error

Supplementary Table S3a. Odds ratios of ocular hypertension according to systolic and diastolic blood pressures and hypertensive status in men

| Characteristics | No. of cases | Odds ratio (95% confidence interval) | | | |
| --- | --- | --- | --- | --- | --- |
|  |  | Age-adjusted | P_trend_ | Multivariable-adjusted^†^ | P_trend_ |
| Systolic blood pressure, mmHg |  |  |  |  |  |
| <120 | 4 | 1 (reference) | 0.02 | 1 (reference) | 0.07 |
| 120–129 | 11 | 3.24 (1.02–10.3) |  | 2.72 (0.80–9.24) |  |
| 130–139 | 11 | 3.17 (1.00–10.1) |  | 2.74 (0.80–9.33) |  |
| 140–159 | 20 | 3.59 (1.90–16.8) |  | 4.59 (1.43–14.7) |  |
| ≥160 | 1 | 1.40 (0.16–12.7) |  | 1.36 (0.14–12.8) |  |
| Diastolic blood pressure, mmHg |  |  |  |  |  |
| <80 | 23 | 1 (reference) | 0.02 | 1 (reference) | 0.05 |
| 80–84 | 9 | 1.37 (0.63–2.99) |  | 1.36 (0.58–3.18) |  |
| 85–89 | 3 | 0.58 (0.17–1.95) |  | 0.67 (0.19–2.30) |  |
| 90–99 | 10 | 2.48 (1.16–5.30) |  | 2.01 (0.80–5.02) |  |
| ≥100 | 2 | 1.72 (0.39–7.52) |  | 1.83 (0.40–8.42) |  |
| Hypertension^¶^ |  |  |  |  |  |
| No | 15 | 1 (reference) | 0.07 | 1 (reference) | 0.21 |
| Yes | 32 | 1.82 (0.96–3.43) |  | 1.58 (0.77–3.21) |  |

^†^Adjusted for age, smoking status, alcohol intake, diabetes, body mass index, LDL-C level, and central corneal thickness. For systolic blood pressure and diastolic blood pressure, we have additionally adjusted for use of any antihypertensive medication.

^¶^Hypertension was defined as use of any antihypertensive medication, systolic blood pressure ≥140 mmHg, and/or diastolic blood pressure ≥90 mmHg.

LDL, low-density lipoprotein.

Supplementary Table S3b. Odds ratios of ocular hypertension according to systolic and diastolic blood pressures and hypertensive status in women

| Characteristics | No. of cases | Odds ratio (95% confidence interval) | | | |
| --- | --- | --- | --- | --- | --- |
|  |  | Age-adjusted | P_trend_ | Multivariable-adjusted^†^ | P_trend_ |
| Systolic blood pressure, mmHg |  |  |  |  |  |
| <120 | 15 | 1 (reference) | <0.001 | 1 (reference) | <0.001 |
| 120–129 | 12 | 1.92 (0.88–4.20) |  | 1.98 (0.75–5.26) |  |
| 130–139 | 15 | 3.05 (1.44–6.45) |  | 2.06 (0.73–5.81) |  |
| 140–159 | 14 | 3.45 (1.58–7.56) |  | 3.43 (1.24–9.50) |  |
| ≥160 | 12 | 13.4 (5.93–30.4) |  | 12.3 (3.97–38.3) |  |
| Diastolic blood pressure, mmHg |  |  |  |  |  |
| <80 | 44 | 1 (reference) | <0.001 | 1 (reference) | <0.001 |
| 80–84 | 7 | 0.97 (0.43–2.17) |  | 1.03 (0.37–2.87) |  |
| 85–89 | 5 | 1.12 (0.44–2.86) |  | 1.25 (0.36–4.34) |  |
| 90–99 | 6 | 1.50 (0.63–3.55) |  | 1.94 (0.65–5.79) |  |
| ≥100 | 6 | 7.77 (3.14–19.2) |  | 11.7 (3.76–36.6) |  |
| Hypertension^¶^ |  |  |  |  |  |
| No | 32 | 1 (reference) | 0.002 | 1 (reference) | 0.02 |
| Yes | 36 | 2.25 (1.34–3.79) |  | 2.36 (1.17–4.78) |  |

^†^Adjusted for age, smoking status, alcohol intake, diabetes, body mass index, LDL-C level, and central corneal thickness. For systolic blood pressure and diastolic blood pressure, we have additionally adjusted for use of any antihypertensive medication.

^¶^Hypertension was defined as use of any antihypertensive medication, systolic blood pressure ≥140 mmHg, and/or diastolic blood pressure ≥90 mmHg.

LDL, low-density lipoprotein.

Supplementary Table S4. Odds ratios of ocular hypertension according to systolic and diastolic blood pressures, stratified by antihypertensive medication use

| Characteristics | No. of cases | Odds ratio (95% confidence interval) | | | |
| --- | --- | --- | --- | --- | --- |
|  |  | Age- and sex- adjusted | P_trend_ | Multivariable-adjusted^†^ | P_trend_ |
| **Participants not using antihypertensive medication (n = 4673)** | | | | | |
| Systolic blood pressure, mmHg |  |  |  |  |  |
| <120 | 17 | 1 (reference) | <0.001 | 1 (reference) | <0.001 |
| 120–129 | 15 | 1.91 (0.94–3.89) |  | 1.82 (0.78–4.24) |  |
| 130–139 | 16 | 2.68 (1.32–5.44) |  | 2.29 (0.97–5.44) |  |
| 140–159 | 22 | 4.15 (2.11–8.14) |  | 3.61 (1.58–8.22) |  |
| ≥160 | 9 | 7.97 (3.41–18.6) |  | 6.91 (2.39–20.0) |  |
| Diastolic blood pressure, mmHg |  |  |  |  |  |
| <80 | 51 | 1 (reference) | <0.001 | 1 (reference) | <0.001 |
| 80–84 | 7 | 0.74 (0.33–1.65) |  | 0.74 (0.33–1.84) |  |
| 85–89 | 4 | 0.65 (0.23–1.80) |  | 0.64 (0.19–2.15) |  |
| 90-9 90–99 | 11 | 1.97 (1.01–3.84) |  | 1.53 (0.62–3.77) |  |
| ≥100 | 6 | 4.00 (1.64–9.63) |  | 3.72 (1.33–10.4) |  |
| **Participants using antihypertensive medication (n = 2110)** | | | | | |
| Systolic blood pressure, mmHg |  |  |  |  |  |
| <120 | 2 | 1 (reference) | 0.01 | 1 (reference) | 0.20 |
| 120–129 | 8 | 3.73 (0.79–17.7) |  | 2.44 (0.48–12.4) |  |
| 130–139 | 10 | 4.00 (0.86–18.2) |  | 1.73 (0.34–8.84) |  |
| 140–159 | 12 | 4.88 (1.08–22.1) |  | 3.16 (0.57–14.9) |  |
| ≥160 | 4 | 7.92 (1.42–44.0) |  | 2.84 (0.38–21.2) |  |
| Diastolic blood pressure, mmHg |  |  |  |  |  |
| <80 | 16 | 1 (reference) | 0.04 | 1 (reference) | 0.05 |
| 80–84 | 9 | 2.02 (0.88–4.62) |  | 2.57 (0.92–7.18) |  |
| 85–89 | 4 | 1.14 (0.38–3.47) |  | 1.35 (0.35–5.21) |  |
| 90–99 | 5 | 1.88 (0.67–5.27) |  | 3.16 (0.97–10.3) |  |
| ≥100 | 2 | 3.93 (0.83–18.6) |  | 5.58 (0.95–32.6) |  |

^†^Adjusted for age, sex, smoking status, alcohol intake, diabetes, body mass index, LDL-cholesterol level, and central corneal thickness.

LDL, low-density lipoprotein.

Supplementary Table S5. Combined effects of systolic and diastolic hypertension on the prevalence of ocular hypertension^¶^

|  | | **Systolic blood pressure, mmHg** | | |
| --- | --- | --- | --- | --- |
|  |  | <120 | 120–159 | ≥160 |
| **Diastolic blood pressure, mmHg** | <80 | 19/2181  Reference | 46/2264  3.05 (1.68–5.54) | 2/24  17.4 (3.51–86.2) |
|  | 80–99 | 0/82  - | 35/1914  2.01 (1.09–3.69) | 5/185  3.16 (1.05–9.50) |
|  | ≥100 | 0/0  - | 2/60  2.62 (0.52–13.3) | 6/73  9.41 (3.26–27.1) |

^¶^Cases/numbers are shown in the upper row, and multivariable-adjusted odds ratios^*^ and 95% confidence intervals are shown in the lower row of each cell.

^*^Adjusted for age, sex, smoking status, alcohol intake, diabetes, body mass index, LDL-cholesterol level.

LDL, low-density lipoprotein.
